# Supplementary figures and images for: Sparsity in an artificial neural network predicts beauty: Towards a model of processing-based aesthetics
Source: PLoS Comput Biol. 2023 Dec 4;19(12):e1011703. doi: 10.1371/journal.pcbi.1011703 (PMC10721202; doi:10.1371/journal.pcbi.1011703)

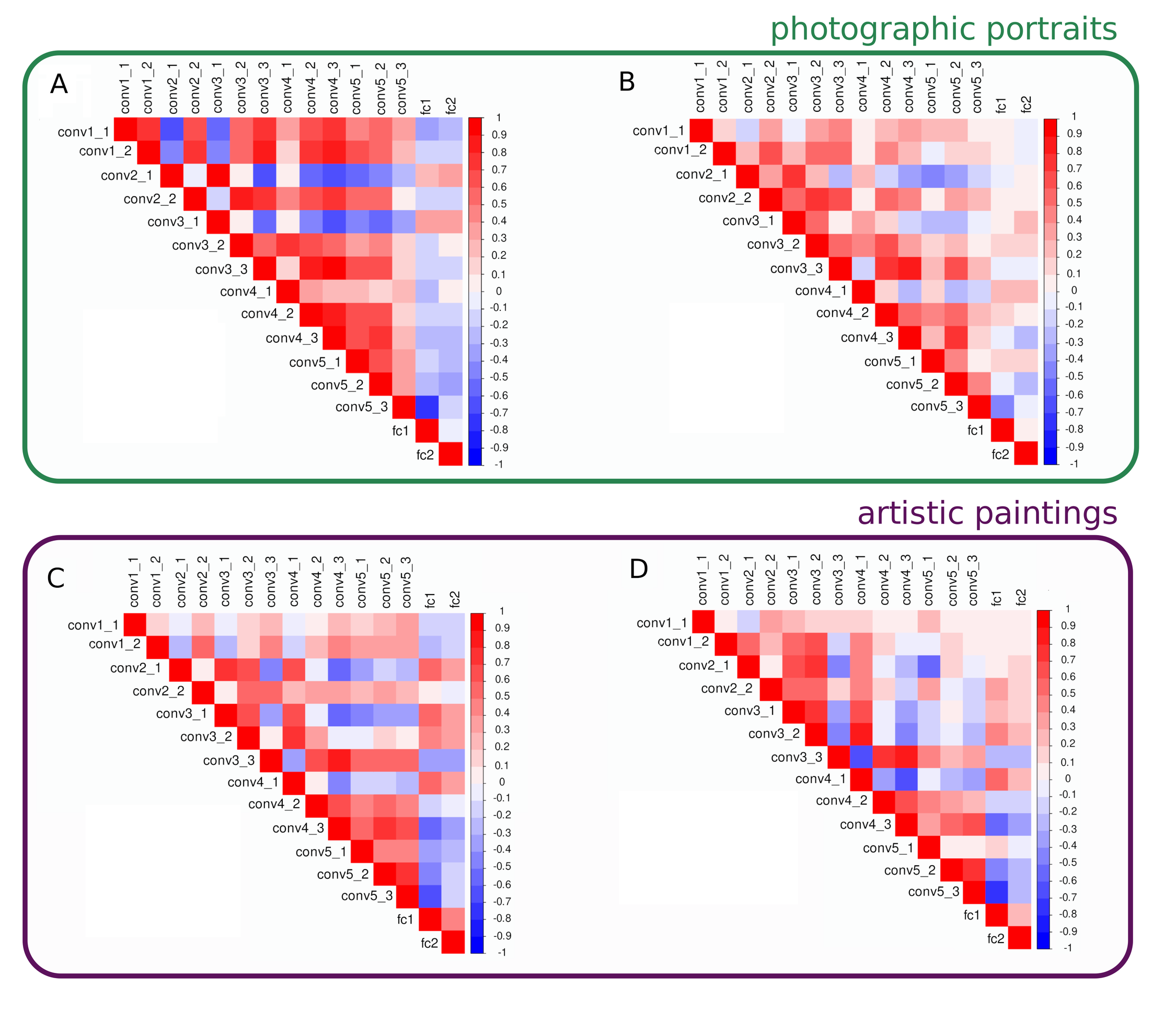

Supplement: S1 Fig — A: CFD dataset. B: SCUT-FBP5500 dataset. C: MART dataset. D: JEN dataset. (TIF) [file pcbi.1011703.s001.tif]

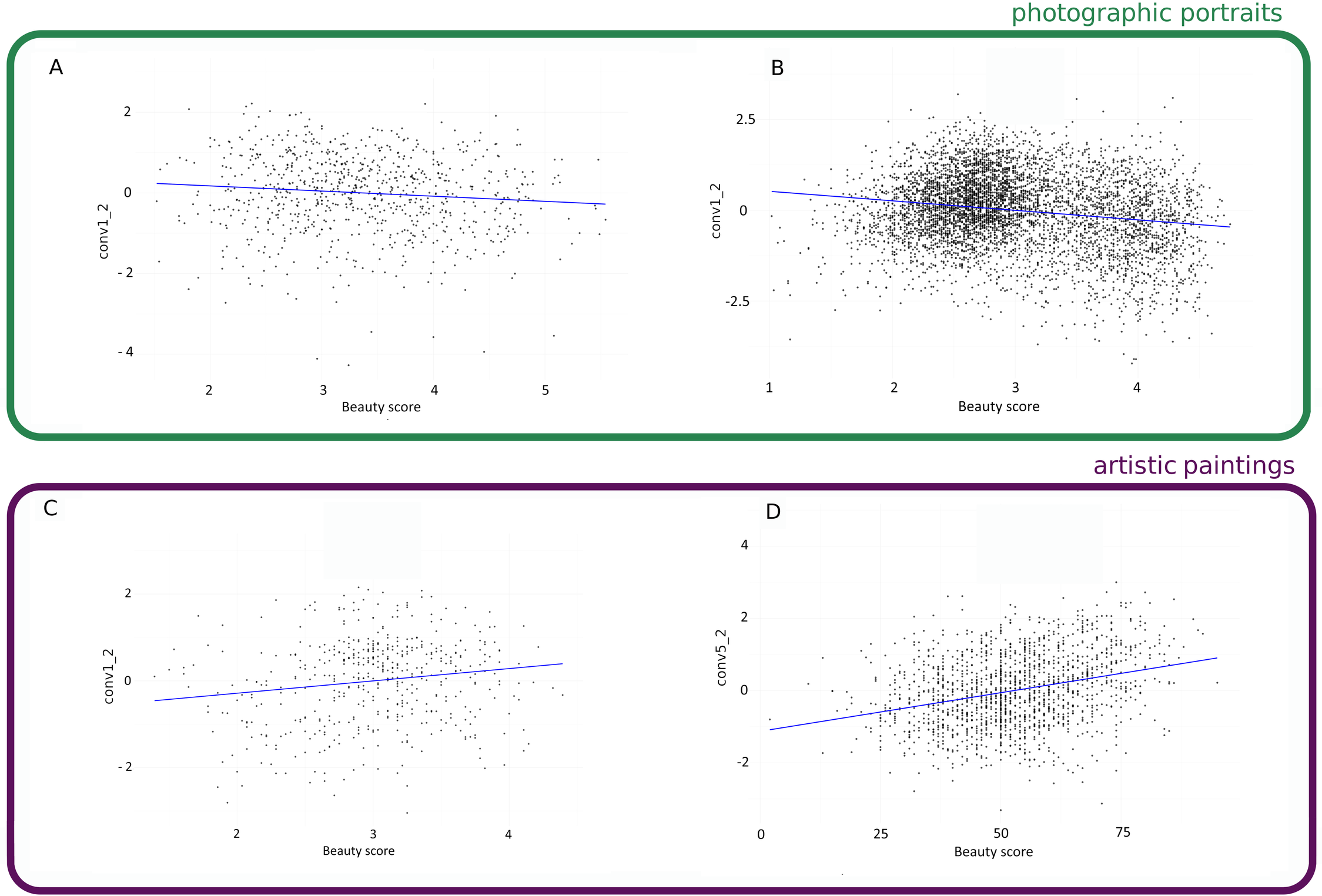

Supplement: S2 Fig — A: CFD dataset (second convolution layer of the first block). B: SCUT-FBP5500 dataset (second convolution layer of the first block). C: MART dataset (second convolution layer of the first block). D: JEN dataset (second convolution layer of the fifth block). The blue trend line corresponds to the predictions provided by the model. (TIF) [file pcbi.1011703.s002.tif]
