# Supplementary material for: Sparsity in an artificial neural network predicts beauty: Towards a model of processing-based aesthetics
Source: PLoS Comput Biol. 2023 Dec 4;19(12):e1011703. doi: 10.1371/journal.pcbi.1011703 (PMC10721202; doi:10.1371/journal.pcbi.1011703)
Supplement: S1 Table — A: CFD dataset. B: SCUT-FBP5500 dataset. C: MART dataset. D: JEN dataset. (DOCX) [file pcbi.1011703.s003.docx]

**S1 Table: Number of PCA components that explained 80% of the total variance of activations for each database and each layer of VGG16.** A: CFD dataset. B: SCUT-FBP5500 dataset. C: MART dataset. D: JEN dataset.

|  | **CFD** | **SCUT-FBP5500** | **MART** | **JEN** |
| --- | --- | --- | --- | --- |
| **conv1_1** | 283 | 1178 | 149 | 745 |
| **conv1_2** | 267 | 1196 | 145 | 749 |
| **conv2_1** | 275 | 1314 | 170 | 844 |
| **conv2_2** | 325 | 1953 | 197 | 911￼ |
| **conv3_1** | 277 | 1502 | 188 | 873 |
| **conv3_2** | 273 | 1656 | 199 | 893 |
| **conv3_3** | 328 | 2058 | 204 | 899 |
| **conv4_1** | 281 | 1771 | 205 | 878 |
| **conv4_2** | 293 | 1844 | 215 | 880 |
| **conv4_3** | 344 | 2162 | 226 | 896 |
| **conv5_1** | 307 | 1779 | 210 | 818 |
| **conv5_2** | 284 | 1565 | 210 | 780 |
| **conv5_3** | 241 | 1256 | 208 | 774 |
| **fc1** | 144 | 452 | 154 | 482 |
| **fc2** | 125 | 330 | 133 | 303 |
