# Supplementary material for: Sparsity in an artificial neural network predicts beauty: Towards a model of processing-based aesthetics
Source: PLoS Comput Biol. 2023 Dec 4;19(12):e1011703. doi: 10.1371/journal.pcbi.1011703 (PMC10721202; doi:10.1371/journal.pcbi.1011703)
Supplement: S2 Table — (DOCX) [file pcbi.1011703.s004.docx]

**S2 Table: Description of evaluators from image datasets.**

|  | **# Evaluators** | **Age** | **Gender** | **Ethnicity** | **Likert scale** |
| --- | --- | --- | --- | --- | --- |
| **CFD** | 1087 | 18-40    mean = 26.75 | 552 females  308 males  227 unknown | 516 White  117 Asian  74 Black  72 biracial or multiracial  57 Latino  18 other  233 did not report | 1 (not attractive at all) to 7 (extremely attractive) |
| **SCUT-FBP5500** | 60 | 18-27  mean = 21.60 | unknow | unknow | 1 (the least attractive) to 5 (the most attractive) |
| **MART** | 100 | 18-65  mean = 39.87 | 74 females  26 males | unknow | 1 (a highly negative emotion) to 7 (a highly positive emotion) |
| **JEN** | 129 | 19-42  mean = 25.30 | 59 females  70 males | 103 germans  26 other | 0 (not beautiful) to 100 (beautiful) |
