# Supplementary material for: Sparsity in an artificial neural network predicts beauty: Towards a model of processing-based aesthetics
Source: PLoS Comput Biol. 2023 Dec 4;19(12):e1011703. doi: 10.1371/journal.pcbi.1011703 (PMC10721202; doi:10.1371/journal.pcbi.1011703)
Supplement: Attachment — Submitted filename: response_to_reviewers.docx [file pcbi.1011703.s005.docx]

Dear editor,

We are very grateful for providing comments from three reviewers for our article entitled: “Sparsity in an artificial neural network predicts beauty: towards a model of processing-based aesthetics”. Following these comments, we have modified our manuscript and detailed these modifications in the text below. In the revised version of our manuscript, we have highlighted in yellow the changes we did

Thank you for your consideration. Please let us know if you require any further information.

Yours Sincerely

Nicolas Dibot

**Reviewer #1:**

**I have reviewed the manuscript titled "Sparsity in an Artificial Neural Network Predicts Beauty: Towards a Model of Processing-Based Aesthetics" by Dibot and co-authors, submitted for publication in PLOS Computational Biology.In this study, the authors present an intriguing investigation on the relationship between the degree of inequality in the hidden layers of a pre-trained convolutional neural network (VGG-16) and the mean opinion scores of beauty in four datasets (two datasets of faces and two datasets of paintings). Specifically, they calculate the Gini index of the activation maps (after RELU) for each image and correlate these values with the beauty measures. When considering individual layers, the R² scores range from approximately 3% to 6%, but considering all layers collectively improves these values to approximately 14% to 28%. Furthermore, the authors conduct regression analyses of principal components associated with activation maps,revealing lower R² scores compared to those obtained with the Gini index, with the exception of abstract paintings. In addition, they explore the combination of principal components with the Gini index, finding no significant improvement in R² scores. The authors adeptly discuss all these findings in the context of the aesthetic theory of fluency.**

**I thoroughly enjoyed reading this work and believe it deserves publication in PLOS Computational Biology.**

We thank the referee for this encouraging comment.

**However, there are a few points I would like the authors to consider before my final recommendation.

The notion that convolutional neural networks accurately emulate the visual representations in the human brain is a subject of debate, and I suggest that the authors acknowledge this limitation of their study.**

We agree it is important to acknowledge this limitation. We have change the last paragraph of the discussion accordingly:

*(l405-420)*

*“Finally, our study relies on both assumptions that DCNNs can model information processing, and that they are valuable tools to study complex mental phenomena. The first assumption has remained controversial [62], as several studies have highlighted differences, some of them significant, between DCNNs and biological vision in the underlying computation (e.g. [63,64]). For example, DCNNs rely on texture information more than shape in object recognition [65]. Yet for many neuroscientists, the emergence in DCNNs of the most fundamental properties observed in animal vision indicates that they are still powerful tools for modelling and understanding this perceptual modality [66,67]. Regarding the second assumption, we acknowledge that purely feed-forward models like VGG16 fall short in describing the full complexity of the judgment of beauty. However, despite top-down cognitive controls that likely influence this judgment, several studies have stressed that it can be made independently of any appeal to cognition[68–70]. Like previous studies showing that VGG16 models reasonably well how the human brain generally processes visual information (e.g., [19]), we concur that the simplicity and tractability of this DCNN can reveal overlooked important brain processes. In particular, it can open a new era in the centuries-old quest to explain and predict beauty.”*

**Alongside AlexNet, VGG16 is one of the initial deep learning models for image classification. Hence, I am curious whether the authors have experimented with different models in their study. Keras provides various pre-trained models (**[**https://keras.io/api/applications/**](https://keras.io/api/applications/)**), and I believe that considering different models would strengthen the authors' conclusions.**

We thank the referee for this suggestion. Testing and comparing different models is indeed relatively easy in Keras. However, we chose to present results with VGG only because it is arguably the most frequently used model on computational neuroscience and the one that has been the most extensively compared to biological vision*.*

**The sentence "at least one layer with a moderate to high R² score" followed by the numbers in brackets might surprise most readers. And that made me wonder about the nature of the association between the GINI and the beauty scores. Is it possible to include at least one example of this association (maybe in SI)?**

To address this comment, we have provided plots illustrating the association between Gini and beauty scores for the layer with the highest R2 in each dataset, in Supplementary Information (see Figure S2.). We have also deleted the word “high” in the sentence highlighted by the referee, which thus now reads*:*

*(l197).*

*"at least one layer with a moderate R² score"*

**Additionally, because beauty scores are very discrete in some datasets, would it make sense to frame the association between sparsity and beauty as a classification problem (maybe for future studies)?**

We thank the referee for this thoughtful comment. Classification models would not consider the ordination of categories (i.e that “score 1”<”score 2”<”score 3”), and thus would be less powerful than linear regression models. However, we acknowledge that the data could be analyzed using ordinal regression modeling. Here, some of the scores (notably those that originally ranged from 0 to 100) were truly continuous, explaining why they were analyzed using a linear regression, assuming that discrete scores (those ranging from 1 to 5) are also continuous variables. We thank the referee again for this suggestion and will consider ordinal regression in our future studies*.*

**I must confess that I was unaware of the utilization of the Gini index as a measure of sparsity. I was further surprised when reading Reference [49] and learning the numerous advantages of this measure. Hence, I suggest that the authors provide more contextual information to justify their excellent choice**.

As suggested, we have added a justification of the Gini index as a measure of sparsity in the revised manuscript:

(l469-477)

*“One study evaluated the ability of various metrics of sparsity to satisfy the attributes desired to properly measure inequity of distribution [72]. The authors found that only the Gini index meets all the desired attributes. For example, kurtosis and L_1_-norm, two other commonly used metrics of sparsity, fail to predict an increase in inequity when adding null values and extremely high values, respectively. The authors did not include the Treves-Rolls metric[73] in their comparison, despite its popular use in neuroscience. We thus used the Gini index and the Treves-Rolls metrics to measure sparsity. The two metrics yielded qualitatively similar results, thus only results with the Gini index are presented here.”*

**On a minor note, please replace "R2" with "R²".**

This has been corrected in the revised version of the manuscript.

**Reviewer #2:**

**This study investigates the link between diverse subjective scores ("beauty" scores) for images and the sparsity of their representation in a conventional VGG-16 deep neural network that was trained for object recognition on the ImageNet dataset. Specifically, the authors study how much of the variance in the subjective scores can be explained by the sparsity of feature responses of different layers of the neural network. To measure the sparsity of images of faces (2 datasets), of abstract paintings (1 dataset) and figurative paintings (1 dataset), the Gini coefficient is calculated for each layer separately and for all layers. For this purpose, appropriate single and multiple regression models are developed. Results show positive and negative correlations between the ratings and the coefficient. R2 values varied widely between and within datasets. Overall, there is no clear-cut answer to the initial question of what the relation between ratings and sparsity might be, except to say that such correlations can indeed be found.**

**The study is interesting, timely and the analysis is valid overall (however, see Point 5 below). I do see some weakness in the initial hypothesis and in the interpretation of the data. Given the ambiguous results, some of the interpretations seem overelaborate and a bit fuzzy.**

**Major points**

**(1) Line 163-164, "empirical score of beauty": The authors use subjective ratings from four different previous studies. They summarily refer to these ratings as "beauty" ratings. A justification of this usage of the terms is needed. (i) In the two datasets of face images, face attractiveness has been rated, which is fine and well-investigated. (ii) In the MART dataset, positive/negative emotions were rated. How emotions relate to beauty perception is not explained in any detail. Paintings that evoke negative feelings, such as sadness or disgust, can nevertheless be beautiful. Can the authors justify their interpretation of emotional ratings as beauty ratings? (iii) For the JenAesthetics dataset, both ratings of "beautiful" and „aesthetic quality" are available. The authors apparently chose to analyze the "aesthetic" ratings, but they refer to them as beauty ratings. Can the authors give a rationale for this? Moreover, the beauty of a painting (i.e. of the formal image composition) is not the same as the attractiveness of faces depicted in an image (see, e.g., Schulz et al., 2017, doi.org/10.3389/fpsyg.2017.02254). There are famous examples of ugly (unattractive) faces depicted in beautiful paintings (e.g., "The Ugly Duchess" by Quentin Matsys). Given these discrepancies, I wonder whether the ratings from the four datasets can really be compared in terms of "beauty" ratings at all. This issue is important because different ratings depend differentially on image properties in general. The heterogeneity of the rating terms may be one of the reasons for the inconsistent results obtained for the four datasets.**

We thank the reviewer for this important comment. We have now added a entirely new paragraph at the end of the introduction explaining the rationale for analyzing different datasets that encompass different visual domains and with different types of beauty ratings:

*(l139-154)*

*“We compared results when analyzing various objects (non-artistic portrait photographs, figurative and abstract artistic paintings) and associated judgments related to beauty (beauty itself, attractiveness, negative-positive emotion) in order to qualitatively explore how generalizable the link between sparsity and beauty is. Beauty is ubiquitous, and can potentially describe any stimulus from natural landscapes to abstract representations, the physical aspect of a face or the artistic merit of its representation [34,35]. Moreover, fluency can mediate the evaluation of beauty in all three brain activities involved in aesthetic experiences: perception, cognition and emotions [36–38]. However, while aesthetic experiences can be both positive or negative (e.g., implying sadness or vertigo; [39]), like other authors we consider beauty as a subset of only positive, hedonically marked aesthetic experiences [5]. This distinction recognizes that some artworks, for example, can have high aesthetic value while being not beautiful. We thus predict that high sparsity, describing elevated fluency, is associated with high scores of beauty across visual domains and judgements of beauty.”*

We have also added a sentence in the Methods section to justify the interpretation of positive emotions in the MART dataset as elevated beauty:

*(l443)*

*“Previous studies have shown that beauty is mostly associated with positive emotions (e.g., Schindler et al. 2017)*

Last, we have corrected the wrong statements that we used “aesthetic” ratings of the JEN dataset: we confirmed that we used “beautiful” ratings (as was indicated in Table 2 in the previous version of manuscript).

**(2) Fluent/sparse/efficient coding, lines 130-142: According to the authors, fluency can be modeled through the concept of efficiency, which is an interesting idea. They state that "a sparse code in one in which the vast majority of neurons are at rest and only a few … neurons are strongly activated". The authors go on to cite studies showing that high sparsity is associated with high attractiveness/beauty of faces. From the studies cited by the authors, the hypothesis would be that higher sparsity is associated with higher beauty of the stimulus. However, a very sparsely coded stimulus would be an empty white sheet with just one short line on it. Would this stimulus be highly beautiful? If this does not fit the hypothesis, please explain more clearly.**

The answer to this classical comment on the link between sparsity and beauty is twofold. First and foremost, it is crucial to differentiate between sparsity and simplicity. From a neuronal perspective, what determines the sparsity is the selectivity of neurons, that is, the range of features that activate a given neuron. This range can be very limited, as in the early stages of the ventral pathway of visual processing or in the first layer of a DCNN, or very large, as in the late stages of the pathway or in the deepest layers of a DCNN. For example, Chang & Tsao (2017) have shown that coding facial identity requires the activation of only ~200 neurons in the inferotemporal cortex. In other words, a complex stimulus (e.g. an object as a whole) can be very sparsely encoded if it activates a single neuron that has been tuned to this object specifically. It is therefore not possible to interpret any association between beauty and sparsity when sparsity is integrated over multiple layers of the DCNN (as in our global ridge regression).

Second, the design of natural and artificial objects is constrained by their functionality, which also contributes to attractiveness. For example, we agree with the referee’s comment that, based on its encoding in the primary visual cortex (as modeled in Renoult et al. 2016 and Holzleitner et al. 2019), a blank face with no eyes and no mouth is more sparse than a normal face. Such a face would not be rated as attractive, probability because it would be perceived as functionally deficient. Most likely, it would also not be rated as beautiful, because of the “misattribution of the fluency effect” (Oppenheimer 2008; Winkielman et al. 2006). Misattribution describes the fact that people are usually unaware of the source of pleasure and, by default, tend to attribute it to the stimulus rather than to information processing itself. Thus, functional deficiency and more generally any kind of object affordance, usefulness or quality influences ratings of beauty by people. However, according to the fluency-base theory of beauty, this influence is a psychological artifact and is not related to beauty strictly speaking. Note that this view of beauty is not a farfetched interpretation of beauty by psychologists, but is strongly rooted in philosophy of aesthetics. To quote Francis Hutcheson, for example: "the pleasure [of beauty] does not arise from any knowledge of principles, proportions, causes or of the usefulness of the object; but strikes us at first with the idea of beauty” (Hutcheson 1725).

The distinction between sparsity and simplicity has been discussed in the cited articles (Renoult et al. 2016 and Holzleitner et al. 2019). Because of space limitation, and to prevent the reader of this interdisciplinary article from getting lost in concepts of psychology that we think are non-essential for understanding the article, we prefer not to dwell on the distinction between beauty and utility (For the reviewer’s record, this distinction is thoroughly discussed in recently submitted review article entitled “Are sexual ornaments beautiful?”).

Chang, L., & Tsao, D. Y. (2017). The code for facial identity in the primate brain. Cell, 169(6), 1013-1028.

Holzleitner IJ, Lee AJ, Hahn AC, Kandrik M, Bovet J, Renoult JP, et al. Comparing theory-driven and data-driven attractiveness models using images of real women’s faces. J Exp Psychol Hum Percept Perform. 2019;45: 1589–1595.

Hutcheson F (1725) An inquiry into the original of our ideas of beauty and virtue (edited and with an introduction by Wolfang Leidhold). Liberty Fund, Indianapolis, 2008

Oppenheimer D.M. 2008 The secret life of fluency. Trends in Cognitive Sciences 12, 237-241.

Renoult JP, Bovet J, Raymond M. Beauty is in the efficient coding of the beholder. R Soc Open Sci. 2016;3: 160027.

Winkielman P., Halberstadt J., Fazendeiro T., Catty S. 2006 Prototypes are attractive because they are easy on the mind. Psychological Sciences **17**, 799-806.

**Line 333-334: "beauty is determined mostly by ease of information processing" (i.e., fluency). Elsewhere, fluency is operationalized as sparse/efficient processing. The results from the present study (negative coefficients in Figure 1) directly contradict this central hypothesis, at least in part. Does this result contradict the fluency theory of beauty perception (see also references 41 and 42)?**

**In conclusion, what I am missing is a clear hypothesis or experimental question that relates to previous research in the field and is tested in this research. With an open question at the beginning (lines 156-158) and ambiguous results at the end, it is not easy to understand what we can learn from this study about the link between beauty, sparsity and efficient coding in the visual system (other than that the relation is a complex one).**

In the revised version of the manuscript, we have clarified that the main goal of the study is to test the hypothesis that considering how information is processed (processing-based aesthetics), in addition to the type of information being processed (feature-based aesthetics) improves prediction of beauty

*(l302-304)*

*“Here, we hypothesized that the way information is processed (processing-based aesthetics), more than the type of information being processed (feature-based aesthetics), drives the evaluation of beauty”*

Moreover, we have now clearly presented that we also predict that processing efficiency (sparsity) should be positively correlated with beauty, across visual domains and type of beauty ratings

*(l138-147*)

*“In this work, analyzing images of various objects, we test the hypothesis that their beauty can be predicted from the sparsity of their DCNN activations. We compared results when analyzing various objects (non-artistic portrait photographs, figurative and abstract artistic paintings) and associated judgments related to beauty (beauty itself, attractiveness, negative-positive emotion) in order to qualitatively explore how generalizable the link between sparsity and beauty is. Beauty is ubiquitous, and can potentially describe any stimulus from natural landscapes to abstract representations, the physical aspect of a face or the artistic merit of its representation [34,35]. The fluency theory posits a unity of the experience of beauty across these domains, driven by a common biological mechanism: the efficiency of information processing.“*

The abstract, part of the results and the beginning of the discussion have also been reworded to stress that our main hypothesis is validated, but not the predictions.

In order to help the uninitiated reader to gradually grasp the concept of fluency, we prefer to formulate the prediction based on the vast majority of predictions and results in the literature on fluency (that is, a positive correlation with beauty). We choose to discuss the studies (more recent and fewer in number) showing that the relationship is not always positive in the discussion section only, as these studies provide possible explanations for the non-validation of our predictions. This choice also allows us to maintain a certain balance in the structure of the article, considering that we have already added more than two paragraphs to the introduction section.

**(3) The review of previous work by other researchers deserves some more attention, especially in the Introduction section. For example, deep neural networks or CNNs have been used extensively before to predict subjective ratings of beauty, in particular, of faces and photographs, and this should be acknowledged in the Introduction, together with appropriate references. The idea to characterize artworks with low-level CNNs features is also not novel (for example, Brachmann et al., Front. Psychol. 2017, 8:830; Iigaya et al., 2021; see general review on AI and art by Cetinic and She, 2022, doi.org/10.1145/3475799).**

We have now included a new paragraph in the revised manuscript presenting some of the previous works using CNN to study beauty.

*(l126-137).*

*“Previous studies in aesthetic science have used DCNNs (for reviews, see [26] and [27]), for example to recognize artworks and classify artistic paintings by their style (e.g.,[28]). DCNNs are also able to predict mean (e.g., [29]) or individual [30] opinion scores of beauty, when trained to do so. Features extracted by DCNNs has permitted the elaboration of feature-based metrics used to unravel the determinants of beauty [31,32]. One study has further shown that artistic and non-artistic images can be distinguished by variance of neuronal activations across layers of a DCNN [26]. Although the authors did not explicitly refer to DCNNs as models of human vision, the variance of neuronal activations describes a state of the visual system while processing an image, and is thus a processing-based metric. Variance, however, is only loosely related to sparsity, and has no direct link to processing efficiency [33]. The use of DCNNs to measure processing efficiency and model fluency thus remains to be investigated.”*

**Moreover, a link between sparse (efficient) coding and beauty perception has been postulated before (see the references in previous papers by the senior author on this topic).**

As suggested, we have cited Christoph Redies’ work in the revised manuscript:

*(l100, 102)*

*“Several authors have argued that fluency could be modeled through the concept of efficiency, which describes the capacity to perform a task with an optimal use of neuronal resources (Redies, 2008; Renoult & Mendelson, 2019)”*

**Lines 102-109: Insert reference to S. Zeki, a prominent proponent of this idea**.

Reference to S. Zeki has been inserted in the revised version of the manuscript:

*(l71)*

*“Beauty is neither a property of objects nor an idiosyncrasy of the observer, rather it emerges from the interaction between the two (Ishizu, Zeki, 2011)”.*

**(4) Lines 127-129 and 320-322. "Processing-based metrics … describing the dynamics of information processing.": I am not sure whether I understand this point correctly. What does "dynamic" refer to in terms of information processing? To this reviewer, "dynamic" implies a change or the production of movement of some sort. With the exception of the "aha effect" (lines 118-120), none of the analyses in this manuscript are carried out as a function of time or imply change, but they refer to static properties extracted from the stimuli and models at one point in time. Also, the ratings analyzed do not change over time in the present study. Please clarify what is "dynamic" about your analyses.**

We thank the referee for this constructive comment about the terminology used. We agree that the term “dynamic” was misleading given that sparsity, as measured here, does not imply any change in the system over time. We thus deleted this term, or replaced it with “efficiency” when appropriate. This involved many small changes throughout the manuscript; here are four examples:

**(a)** *(l51-53)*

*“Recent theories in psychology emphasize the importance of information processing dynamics”*

has been replaced by

*“Recent theories in psychology emphasize the importance of considering how the brain processes features, in addition to the features themselves”.*

**(b)** *(l202-205)*

*“people's perception of beauty is most influenced by the dynamics of processing texture or local color contrasts, while for figurative paintings, the dynamics of processing objects as a whole, and the spatial arrangement of their parts, is more important.”*

has been replaced by

*“people's perception of beauty is most influenced by the efficiency of processing texture or local color contrasts, while for figurative paintings, the efficiency of processing objects as a whole, and the spatial arrangement of their parts, is more important.”*

**(c )** *(l304-307)*

*“We thus investigated whether visual beauty could be predicted from the sparsity of neuronal activations in an artificial convolutional neural network, which characterizes the dynamics of processing visual stimuli independently of the features describing these stimuli.”*

has been replaced by

*“We thus investigated whether visual beauty could be predicted from the sparsity of neuronal activations in an artificial deep convolutional neural network (DCNN), a statistic characterizing the efficiency of processing visual stimuli that is independent of the features describing these stimuli.”*

**(d)** *(l309-311):*

*“information processing likely contributes more than features to explaining variation in perceived beauty”*

has been replaced by

*“information processing itself likely contributes more than the processed features to explaining variation in perceived beauty.”*

**Also, the difference between "feature-based" aesthetics and "processing-based" aesthetics is not evident to this reviewer. Perceptual features are processed by the visual system, too. What does the low-level visual system process other than image features? Like variance (Brachmann et al., 2017), sparsity is a measure of the global distribution of activation in the network layers. Perhaps derive a term from that definition somehow?**

We fully agree that how "feature-based" and "processing-based" metrics were presented and defined was misleading. We have kept these expressions in the revised manuscript, but have more clearly defined them:

*(l93-99)*

*“Modeling beauty using the fluency theory thus requires developing metrics that describe the state of the sensory system or the brain while it is processing information. We qualify such metrics as “processing-based”. In contrast, “feature-based” metrics are focused on features, which are quantitative characteristics of objects, measured either directly on the object or as they are perceived. Contrary to processing-based metrics, which are interactive by construction, feature-based metrics can thus be either interactive or objective.”*

**(5) Lines 501-505: The authors carried out 10-fold cross-validation to obtain R2 scores. With small sample size, these scores can be unstable potentially. The authors should consider repeating the cross-validations many times randomly and then calculate and compare the averages obtained from the n-times 10-fold cross-validations. The number of repetitions n should be high enough for testing whether the means are significantly different from 0. This is especially important for data with small R2 values and from small datasets (e.g., the JenAesthetics dataset). If you already did this or something similar, please clarify.**

We had indeed carried out 10-fold cross-validation repeated 10 times, but had not made this clear in the text.

In the revised version of the manuscript, we have nevertheless reproduced these analyses with 100 repetitions to ensure stability: this did not alter the results. We have also stated more clearly that we averaged the R2 scores over 100 repetitions.

*(l507-512)*

*“For all linear regression and linear Ridge models, we performed a 10-fold cross-validation, repeated 100 times to ensure that the results were stable, calculating the coefficient of determination (R^2^ score for univariate models and adjusted R^2^ score for multivariate models) between predicted and empirical beauty scores for each test fold, and then averaging the R^2^ score over the 10 folds and then over the 100 repetitio­­­ns. “*

**(6) The authors use Gini's coefficient as a measure for uneven distribution or inequality between the measured activations of the CNN features, which is an interesting and valuable idea. Another measure of inequality of values would be variance. Brachmann et al. (2017) studied different types of variance of CNN filters previously. Their results suggest that traditional artworks are richer (i.e., less sparse!) than various types of non-art images at low CNN levels. This result is opposite to the authors' hypothesis of a positive correlation between sparseness/fluency and beauty. The authors may consider discussing this contradiction.**

We thank the referee for this suggestion. Brachmann et al. (2017) explored whether art and non-art images could be discriminated against based on metrics describing the variance in CNN activations. The authors investigated different metrics, and combinations of metrics, all based on variance. Although the authors interpret one of their most discriminant metrics (the mean overall variance of all filter responses) in terms of sparsity, assuming that high variance describes high sparsity, the relation between variance and sparsity may be more complex.

First, sparsity can vary independently of variance. This has been well-studied in neuroscience, for example in Tolhurst et al. (2009, compare Figure 3E and 3A).

Second, in the context of CNN, we expect a negative, rather than positive, relation between sparsity and variance. This type of relation is indeed observed with most metrics of sparsity (e.g., kurtosis, Gini index, L1-norm). For Gini index and L1-norm, in particular, this relation is due to the zero-inflated distribution of neuronal activations. Indeed, setting many activations to zero (i.e., increasing sparsity) removes the contribution of these values to the calculus of variance, and thus decreases variance. Rather than an artifact, this behavior is a desired property of metrics of sparsity (see Hurley and Rickard 2009, and Rickard and Fallon 2004 for Gini index specifically). It notably explains the effectiveness of the L1-norm regularization (e.g, in Lasso regression) which, by increasing sparsity in regression coefficients, decreases the variance of these coefficients, and thus the risk of overfitting. Crucially, the distributions of activations in most CNNs (including AlexNet, used in Brachmann et al. (2017), and VGG16, used in our study) are zero-inflated because of the ReLU layer that follows each convolution layer (ReLU sets all negative values to zero). In CNNs, therefore, high variance in neuronal activations is generally associated with low sparsity.

Because Gini index and variance are not obviously related to one another, we view them as distinct metrics, measuring different statistics of CNN’s activations.

Moreover, Brachmann et al. (2017) made a very interesting contribution in comparing artwork and non-artwork images, but they did not use their variance-based metric to compare visually attractive or beautiful images and unattractive or less beautiful images (either of artwork or non-artwork). In a previous comment, the reviewer himself stressed that not all artworks are beautiful. Some artists even claim to make “a-beautiful” art. For several authors (e.g, Brielmann and Pelli 2008; Brown and Dissanayake 2009), conflating the study of art with that of beauty and aesthetics is a major current limitation of the field of empirical aesthetics.

For all these reasons, we preferred not comparing in depth our results to those of Brachmann et al. (2017) in the discussion section. Nevertheless, we have presented the results of Brachmann et al. in the introduction as an example of processing-based metrics derived from CNNs, simply highlighting that variance and sparsity are distinct metrics

Brachmann, A., Barth, E., & Redies, C. (2017). Using CNN features to better understand what makes visual artworks special. Frontiers in psychology, 8, 830

Brielmann, A. A., & Pelli, D. G. (2018). Aesthetics. Current Biology, 28(16), R859-R863.

Brown, S., & Dissanayake, E. (2018). The arts are more than aesthetics: Neuroaesthetics as narrow aesthetics. In Neuroaesthetics (pp. 43-57). Routledge.

Hurley N, Rickard S. Comparing Measures of Sparsity. IEEE Trans Inf Theory. 2009;55: 4723–4741. doi:10.1109/TIT.2009.2027527

Rickard, S. & Fallon, M. (2004) “The Gini index of speech,” Conference on Information Sciences and Systems, Princeton, NJ.

Tolhurst, D. J., Smyth, D., & Thompson, I. D. (2009). The sparseness of neuronal responses in ferret primary visual cortex. Journal of Neuroscience, 29(8), 2355-2370.”

**(7) The beginning of the Discussion section (lines 318-356) is too long and has review-like passages. It should be shortened considerably. By contrast, the rest of the Discussion (lines 357-418) focuses on what has actually been found (or not) in the present study and interprets the findings in relation to previous studies. This part of the Discussion provides a much more balanced view of the different theories in the field than the Introduction section. Consider moving some of this into the Introduction (see Point 2). For example, you mention that you refrain from comparing results across datasets (line 362). That sounds a bit different in the Introduction and Results sections where you do compare results between datasets. Because the Introduction sets the stage for the Results, the major restrictions of the study, such as the small number of datasets and different "beauty" scores (see above), should be made clear already at the beginning of the paper.**

Following the reviewer’s suggestion, we have rewritten the beginning of the discussion, and notably the second paragraph (l304-314). We have also clarified the fact that the main goal of studying different datasets is to evaluate the generalization of the finding, but we also acknowledge that this study is preliminary and that further studies are needed to explore more datasets, and to investigate intra- and inter-domain variations in the link between processing and beauty (l327-333). In the introduction, we have now clearly written that the comparison across datasets is qualitative:

*(l139-143)*

*“We compared results when analyzing various objects (non-artistic portrait photographs, figurative and abstract artistic paintings) and associated judgments related to beauty (beauty itself, attractiveness, negative-positive emotion) in order to qualitatively explore how generalizable the link between sparsity and beauty is”*

**Minor points**

**(8) Lines 223-227 and 251-253: The authors did not measure micro-experiences of fluency in their study. This is an interpretation that would be more appropriate for the Discussion section.**

We preferred to keep the first citation of micro-experiences of fluency unchanged because it is necessary for the reader to understand the rationale of the analysis. However, in the second citation, we have clarified that we are not measuring micro-experiences:

*(l241-244)*

*“Yet, postulating that the multivariate regression provides a model of how fluency micro-experiences aggregate, our results suggest that this aggregation could be more complex than a mere sum of micro-experiences and may rather involve top-down controls weighting their importance in different stages of information processing”*

**(9) Figures 1, 2, and S1: Some of the lettering is barely visible on a regular print-out.**

*We thank the referee for noticing this. We modified the figures accordingly.*

**(10) The present version of the code which the authors kindly provided at the Github site contains uncommented code from other experiments. A code restricted to the present experiments and annotated with more explanations would be helpful for users who want to understand the processing of the data in detail.**

*As suggested, we have better explained the code with more exhaustive comment sections. A description has been added at the beginning of each code file to describe the analyses that are performed specifically in that file. There were also some superfluous functions that did not correspond to analyses presented in the article: these have been removed for greater clarity. Finally, the "README" files have been completed to describe the content of each file more precisely.*

**Reviewer #3:**

**This is definitely an interesting article addressing the problem how to model beauty of different kinds, e.g. artworks, faces etc. The authors claim that neuronal sparsity correlates with beauty scores, explaining up to 28% of their variance and up to 47% when combined with a feature-based metric.**

**Their concept of sparsity is strongly referring to fluently processed visual stimuli. Later, they critically reflect on this model, which was put forward by the so-called hedonic fluency model, a very established model of aesthetic appreciation.**

**Importantly, they propose that it is not about the object as such, which is clever, but that beauty aspects arise by the interaction of the perceiver and the object; and fluency in this respect seems to be an ideal theoretical framework to model such interactive components. But here is also the fundamental problem of the highly appreciated approach provided by the authors: Fluency can, of course, be measured by the average speed of processed pictures, but much more interesting is the experimental treatment and manipulation of fluency and so how the neural network as a representative model would react on such increased familiarity and so fluency.**

We thank the referee for this thoughtful comment. In our study, we indeed proposed to model fluency through the efficiency of the neural code, measured as the sparsity of neuronal activations. Thus, fluency is modeled when considering both the model of perception (CNN) and the stimuli (the images) at a given time stamp, without considering any effect of manipulating either the model of perception or the stimuli. Such manipulations are very interesting extensions of the proposed approach; however, we think they would require publication in separate articles. In the discussion of revised manuscript, we have included a sentence mentioning the interest of such manipulations for future studies:

(l336-342)

*“Regardless of the level of overlap between typicality and sparsity, both [34] and this study considered a static perceptual system. In contrast, a classical approach to study fluency in psychology is to investigate how the system varies when manipulating fluency, for example using matching prime (see [36]). In the future, it would thus be interesting to apply similar dynamic approaches to DCNNs, for example by analyzing how progressively tuning the DCNN to features that are specific to the target stimuli increases the ability of sparsity to explain beauty”*

Note that, besides this study, we are currently preparing another article in which we are comparing the ability of different CNN-based metrics of fluency to predict facial beauty. One of these metrics is based on the likelihood of a stimulus estimated at different layers of the CNN, an approach inspired by Ryali, C. K., Goffin, S., Winkielman, P., & Yu, A. J. (2020). From likely to likable: The role of statistical typicality in human social assessment of faces. Proceedings of the National Academy of Sciences, 117(47), 29371-29380. Here, the likelihood is estimated from a reference distribution of features (a probability density function). Notably, we investigated how the level of specialization of the reference distribution influences the ability of likelihood to predict beauty: for example, we predicted that likelihood would explain the facial beauty of Asian males better when the reference distribution depicts Asian males only, compared to when it depicts either Asian or male people, and compared to when it depicts both males and females of all ethnic groups. Specializing the reference distribution is equivalent to increasing the familiarity of the perceptual space with the evaluated stimuli. We found that the level of specialization indeed influences the results in the predicted direction, but also that, whatever the level of specialization, likelihood was a less good predictor of beauty than sparsity (as measured in this study) is.

In the near future, we plan to explore the idea of manipulating the familiarity of the CNN with the target stimulus even further, e.g. by manipulating the sensitivity of artificial neurons (rather than the shape of the perceptual space through the probability density function) using matching and mismatching primers (as Exp 1 of Reber, R., Winkielman, P., & Schwarz, N. (1998). Effects of perceptual fluency on affective judgments. Psychological science, 9(1), 45-48.),

Last, we have previously tried to also model fluency through the computing time (time needed for a CNN to process an image); however, with regular computer devices we found this measure to be unreliable, too dependent on external factors (e.g., heat of the machine).

**Besides this fundamental criticism, I really liked the general approach. I have found only the following aspects which should be addressed:**

**Winkielman instead of Winkelman**

We thank the referee for noting this spelling mistake, which has been corrected in the revised version of the manuscript (l105)

**all statistical parameters should be italicized, e.g., p and R**

This has been corrected in the revised version of the manuscript.

**R2: 2 should be superscripted**

This has been corrected in the revised version of the manuscript.

**[42]: there exists an interesting addition: Carbon, C. C., & Albrecht, S. (2016). The Fluency Amplification Model supports the GANE principle of arousal enhancement. Behavioral and Brain Sciences, 39.**

We thank the reviewer for pointing out this reference. We have included a sentence referring to this article in the revised manuscript:

*(l389-393)*

*“One explanation is that fluency would work as an amplifier, being positively correlated with beauty for stimuli with a positive emotional valence, and negatively correlated for negatively-valenced stimuli [42]. This is in line with the general idea that a core goal of perceptual processes is to disambiguate stimuli and thus enable the most appropriate behavior”*

**beauty “as such”: must be made clear what kind of beauty aspects can be covered by the present approach, for instance, NOT extraordinary beauty.**

In order to address this comment, and a related comment raised by Reviewer #2, we have made it clear in the introduction that different facets of beauty are investigated in the different datasets (e.g., physical beauty for the standardized portrait faces of CFD, artistic beauty for paintings of the Jen dataset, aesthetic emotion in the abstract paintings of MART) *(l138-145)*

**other literature on different models should be included to assess the quality of predictive power of the current model against established ones**

We acknowledge the importance of comparing the predictive power of our model with that of alternative models. Such comparisons are meaningful, however, only under restricted conditions. One of these conditions is the comparison of models using the same dataset. Indeed, when we evaluated previously published models, we generally found notable differences between the power presented in the original articles and that obtained with our datasets. For example, the feature-based metrics used in our study (PCA-reduced deep features extracted from VGG16 pretrained on Imagenet + ridge regression) reproduce those of Ligaya et al. 2021. However, unlike this study, which found R2 scores ranging from 0.25 to 0.5 depending on the layers (see Figure 5B in Ligaya et al), when applied to our different datasets, the model yielded significantly lower R2 values (see our Figure 2). Moreover, as explained l166-168, one goal of using a pre-trained CNN is to study biologically realistic features (that is, image statistics that are likely to be encoded by the visual system) - our study thus cannot be compared with AI-based models optimized for beauty prediction. For all these reasons, we have limited comparisons to the study of Ryali et al., who also studied the CFD dataset using a perceptual face space. However, if the reviewer specifically provides us with some references to other models comparable to ours, we would be delighted to discuss them in a future version of the manuscript.
